# Comparative Study on the Composition of Oil Bodies from High-Oleic Peanuts

**DOI:** 10.3390/foods15010177

**Published:** 2026-01-05

**Authors:** Lixia Zhang, Songli Wei, Xiaojing Sun, Xin Lu, Shangde Sun, Runfeng Du, Shanshan Guo

**Affiliations:** 1 Research Center of Agro-Products Processing, Henan Academy of Agricultural Sciences, Zhengzhou 450002, China; lxzhang2003@163.com (L.Z.); weisongli1991@163.com (S.W.); 18300697186@163.com (X.S.); 13700879657@163.com (R.D.); 2 School of Food Science and Engineering, Henan University of Technology, Zhengzhou 450001, China; shangdesun@haut.edu.cn; 3 Henan Qihua Edible Oil Limited Company, Hebi 153000, China; 18939286629@163.com

**Keywords:** oil bodies, cultivar, composition, fatty acids, tocopherols, amino acids

## Abstract

Compositional heterogeneity of oil bodies (OB) from nine high-oleic peanut (HOP) cultivars was systematically characterized. The results demonstrated that nine OB samples exhibited variability in R, G, and B values (red, green, and blue color channels), with the B channel values significantly differing among cultivars, while no significant color variation was observed in their overall appearance. Fats and proteins dominated the dry matter composition of OB, consistent with typical plant OB structural profiles. The high-fat OB of cultivars J572-O, J6-O, Z215-O, and H985-O exhibited outstanding efficiency in loading lipophilic bioactive compounds. OBs from J16-O, G37-O, Z215-O, J572-O, Y37-O, and Y65-O had a distinctive fatty acid profile: high-oleic acid and monounsaturated fatty acids (MUFAs), with reduced linoleic acid, palmitoleic acid, and saturated fatty acids (SFAs). All OB samples contained four tocopherol isomers (α-, β-, γ-, δ-), with α-tocopherol (5.07–12.59 mg/100 g) and γ-tocopherol (6.36–14.81 mg/100 g) as the predominant forms. Essential amino acids (EAAs) and hydrophobic amino acids were detected, with leucine, phenylalanine, and valine being highly abundant. TEAA/TAA and TEAA/TNEAA ratios complied with FAO/WHO standards. J16-O stood out with a balanced fatty acid profile, high tocopherols, and quality protein, making it a promising candidate for functional foods.

## 1. Introduction

As an important source of edible oil and protein, peanuts are grown globally and preferred by consumers due to their high nutritional value and unique flavor [1,2]. Peanut oil is rich in various unsaturated fatty acids, while peanut protein is abundant in eight essential amino acids vital for the human body, which attributes make them a widely sought-after plant protein resource in the food industry [2]. While non-flavor factors such as poor frying and oxidation stability remain, the short shelf-life of peanuts caused by high levels of linoleic acid has severely restricted normal peanut development in the food industry. High-oleic peanuts (HOP), newly developed peanut varieties, have superior flavor, quality, and storage properties than normal-oleic peanuts (which typically contain 35–69% oleic acid) and thus are a popular research topic [3,4].

HOP denotes peanuts containing more than 75% oleic acid in their fatty acid composition [5] and an oleic to linoleic acid ratio (O/L) typically greater than nine [6]. These cultivars were developed through conventional breeding techniques, including hybridization and natural mutation screening, and are classified as non-genetically modified organisms (non-GMO). Due to this special fatty acid composition, HOP boosts multiple health functions, such as lowering blood lipid levels, protecting cardiovascular, cerebrovascular, and liver tissue, controlling blood sugar and body weight, delaying aging, and enhancing brain cognitive ability [7]. The oxidation rate of oleic acid is approximately 1/10 of linoleic acid, so both high-oleic acid peanut raw materials and their products show good oxidation stability, which helps processing times and reduces or even avoids waste [8,9]. Peanuts are a good source of tocopherols; the tocopherol content of peanuts varies with variety and production location [10].

The oil in HOP is stored in subcellular organelles called oil bodies (OB), which have good emulsification properties, making it a highly promising natural emulsifier [11]. OB extracted from HOP not only has excellent emulsification but also improved nutritional value and application properties (such as good oxidation stability) compared with normal-oleic peanut OB [12]. Component composition is a key quality trait that affects nutritional and health values, processing characteristics and efficiency, storage resistance, and market competitiveness of peanuts and their processed products [13]; thus, understanding OB composition from HOP is essential. Nevertheless, few relevant studies have been reported, and the comparative analysis of OB among HOP compositions is still unclear.

In this study, nine HOP varieties were selected to comprehensively evaluate differences among OB composition for different HOP varieties by determining fat content, fatty acid composition, protein content, amino acid composition, and other indicators. The results aim to provide theoretical support for the application of high-oleic peanuts OB.

## 2. Materials and Methods

### 2.1. Materials

Nine different HOP cultivars (Y37, Y65, J16, J572, J6, Z215, G37, H985, and K1715) were obtained from the National and Local Joint Engineering Laboratory for Peanut Genetic Improvement. Petroleum ether (boiling range 30–60 °C), concentrated sulfuric acid (95–98%), methanol, boron trifluoride, sodium chloride, n-hexane, anhydrous sodium sulfate, and other chemical reagents were analytically pure and purchased from Tianjin Kaitong Chemical Reagent Co., Ltd. (Tianjin, China). Deionized water (resistivity ≥ 18.2 MΩ·cm) was produced in the laboratory using a Milli-Q water purification system (Millipore, Bedford, MA, USA).

### 2.2. OB Extraction

OB from different cultivars was extracted according to Chen and Ono [14], with minor modifications. Fifty grams of peeled peanuts were soaked in deionized water at a 1:10 ratio (*w*/*v*) for 2 h, then transferred soaked peanuts together with water to a crusher (BM-RE08, Zojirushi Co., Osaka, Japan) for grinding at high speed for 2 min. The homogenate was then filtered through four layers of gauze (100 mesh pore dimensions), followed by centrifugation with a centrifuge (DZ267-32C6, Anting Scientific Instrument Factory, Shanghai, China) at approximately 5500 rpm for 15 min. The top cream layer was gathered and dispersed in a 20 wt% sucrose solution. The pH value of this solution was adjusted to 11.0 with 1 M NaOH. Suspension was stirred for 15 min and centrifuged at 5000 *g*/~5500 rpm for 15 min. The top cream layer was again collected and dispersed in a 20 wt% sucrose solution. The above steps were repeated twice to purify OB, which was obtained by collecting the topmost layer and storing it at 4 °C for the following steps.

### 2.3. Detection of RGB Color 

RGB color mode is the most widespread and basic color measurement mode, which can effectively avoid deficiencies from the naked eye in spectral response and sensory ability, where R, G, and B represent red, green, and blue color channels, respectively [15]. The color of nine fresh peanut OB was evaluated by RGB analysis according to our previous method [11]. Twenty milliliters of a fresh peanut OB was poured into a quartz Petri dish (90 mm) and placed in a fixed position in a closed compartment equipped with a fluorescent lamp with 6 W and a color temperature of 4000 K (white color). The compartment, whose dimensions were 30 cm × 22 cm × 23 cm, was internally lined with white office paper, according to principles of light reflection [16]. A webcam was employed in the compartment for the acquisition of digital images. The recorded images contain 24-bit (16.7 million colors) and 2880 pixels × 1620 pixels spatial resolution, and are stored in JPEG format (jpg). In addition, images were uniformly processed using the RGB color model in Adobe Photoshop CS6 13.0.1 software by computer. To ensure accuracy, three different parts of each image were selected for taking measurements, and the average RGB values of these triplicate images were then used for further analysis.

### 2.4. Analysis of the Basic Composition

To clarify differences in composition among the nine OB, basic OB composition was classified. Low-temperature vacuum drying was used to ensure OB structural integrity. Glass dishes containing 20 g samples were dried in a vacuum drying oven at 70 °C to a constant weight, transferred to a desiccator, and cooled to room temperature, then weighed. Sample moisture content was calculated by weight differences before and after drying.

The OB fat content in dried OB was determined according to the Soxhlet extraction method [17]. Subsequently, the Soxhlet-extracted OB fats were collected and refrigerated at −18 °C to calculate fatty acid composition.

Protein content of the oil body was determined by GK-700 automatic Kjeldahl nitrogen analyzer (Shandong GreenKare Precision Instruments Co., Ltd., Heze, China) [18]. The program was set as follows: 250 °C to 400 °C, 30 min for every 50 °C rise, 2.5 h for the final rise to 420 °C, then gradual cooling to room temperature.

### 2.5. Analysis of the Fatty Acids Composition

Fatty acid analysis of OB fats was performed using gas chromatography (GC, Agilent 7890A, Agilent Technologies, Santa Clara, CA, USA). About 0.1 g of OB fats extracted as described in “2.4” was added into a round-bottom flask to convert fat to fatty acid methyl esters [19]. Methyl esterified fatty acids were then filtered into the injection vial and placed on a GC detector for fatty acid identification. Analytical conditions were followed according to Wei et al. [11]. Then fatty acids were characterized using retention time of fatty acid methyl ester standards (Sigma-Aldrich, Shanghai, China) and quantified by the peak area normalization method.

### 2.6. Determination of Tocopherols Profile

The chromatographic detection conditions were set according to Mei et al. [20]. OB tocopherol content and composition were analyzed using a Shimadzu LC-10A reversed-phase high-performance liquid chromatography (RP-HPLC) (Kyoto, Japan) equipped with a Shodex C18 column (4.6 mm i.d. × 250 mm), connected to an autoinjector and a Shimadzu SPD-10A UV-vis detector (Kyoto, Japan). The mobile phase employed was acetonitrile/methanol/dichloromethane (60:35:5) at a flow rate of 2 mL/min. Vacuum dried OB (3.0 g) was taken in a flat bottom flask and warm water (20 mL), ascorbic acid (1.0 g), butylated hydroxytoluene (BHT, 0.1 g), anhydrous ethanol (30 mL), and potassium hydroxide (20 mL) were then added, followed by mixing and saponification in a constant temperature water bath at 80 °C for 30 min. The saponification solution was transferred to a partition funnel and extracted three times using a petroleum ether–ether mixture (1:1 *v*/*v*). The extract was then concentrated by rotary evaporation, and the residue was dissolved and volumized with methanol. The solution was then passed through a 0.22 μm organic filter membrane to determine high-performance liquid chromatography. Peaks were identified and quantified by comparison with tocols (α-, β-, γ-, δ-tocopherol) used as external standards. The concentration of tocols standard solution was 0.25, 0.50, 1.00, 2.00, 5.00 μg·mL^−1^, and 10.00 μg·mL^−1^, separately.

### 2.7. Determination of Amino Acids Profile

The amino acid composition of the oil body proteins was analyzed using the method previously described by Li et al. [21] and Zhang et al. [2]. Briefly, 20 mg of dried and defatted OB was accurately weighed and transferred into an anaerobic tube containing 10 mL of HCl (6 mol/L) and 3–4 drops of phenol. The tube was sealed after purging with nitrogen for 5 min and then subjected to hydrolysis in an oven at 110 °C for 24 h. The hydrolysate was transferred, allowed to cool, filtered, and diluted to a final volume of 100 mL in a volumetric flask. From the filtrate, 1 mL was evaporated in a test tube concentrator. Subsequently, 1 mL of sample diluent was added, and the mixture underwent ultrasonication for 2 min. Finally, the sample was analyzed using an amino acid analyzer (Agilent 1200 HPLC system, Palo Alto, CA, USA) for amino acid profile analysis equipped with an Agilent ZORBAX RX-SIL column (250 mm, 4.6 mm i.d., 5 μm). The detection wavelengths were 570 nm and 440 nm. The isocratic flow rate of mobile phase n-hexane/isopropyl alcohol (99.5/0.5, *v*/*v*) was 1.0 mL/min, and the column oven temperature was set at 30 °C.

### 2.8. Statistical Analysis

Each experiment was performed three times in parallel. Data collection and analysis were performed by Origin 2019 (Origin lab Software Corp, Northampton, MA, USA). Differences between samples were analyzed by IBM SPSS statistics 23 (SPSS Inc., Chicago, IL, USA), where *p* < 0.05 was considered significantly different and different letters (a–h) indicated statistically different levels.

## 3. Results and Discussions

### 3.1. RGB Color Analysis

RGB color analysis technology is an image processing technology based on computer vision, which is fast, convenient, and nondestructive. Peng et al. [11,22] demonstrate that digital image analysis with RGB parameters can effectively differentiate color variations in emulsion-based samples with similar optical properties to oil bodies. Additionally, the colors of these nine OB were all milky white to the naked eye; they showed differences under RGB analysis (Table 1). Generally, the order for these three-color values of tested samples is G > R > B, except for G37. Moreover, R and G values were not statistically different; there were significant differences among B values, indicating that B values can help initially distinguish the source of OB. To reduce the impact of shadows on images, the three R, G, and B values were normalized to obtain R, G, and B values. The three normalized values for all samples were between 0.3208 and 0.3419. There was no difference in G values (*p* > 0.05), but there were significant statistical differences for R and B values (*p* < 0.05). For each sample, the R (0.3324–0.3406), G (0.3384–0.3419), and B (0.3208–0.3276) values did not show much difference, indicating that red, green, and blue contribute similarly to OB color, whereby colors for all OB were close to white, which was consistent with naked eye observations.

### 3.2. Basic Composition

Basic composition (Table 2) is the fundamental index for evaluating the composition of the tested OB. Moisture content in fresh OB was 18–24.25%, which was higher than previous reports (12.57%) [23]. This outcome may be due to differences in protein content and type, which result in different water binding capacity [24]. Crude fat content for these OB was 76.98–93.22%, similar to fat content in normal-oleic peanut OB [25]. Y37-O showed the lowest fat content among the nine samples and was the only one with fat content less than 80%. Some OB showed high fat content, especially J572-O, J6-O, Z215-O, and H985-O, which contained up to 93.22%. Therefore, some tested OB may be more suitable as a carrier for oil-soluble active substances, depending on their crude fat content. Protein content was a key factor affecting OB emulsification performance. Crude protein content of OB fluctuated between 0.89% and 1.59% for H985-O and G37-O, showing the lowest and highest values, respectively. Except for G37-O and H985-O, most OB showed protein content around 1.45 with no significant difference (*p* > 0.05). Generally, the tested OB contained large amounts of fat and a small amount of protein, which agreed with the composition of peanut OB proposed by Niu and Chen [26,27].

### 3.3. Fatty Acid Composition

Eight fatty acids were quantified among tested OB (Table 3), which containing five saturated fatty acids (SFA: C16:0, C18:0, C20:0, C22:0, C24:0), two monounsaturated fatty acid (MUFA: C18:1, C20:1), and one polyunsaturated fatty acid (PUFA: C18:2). The order of abundance for these fatty acids was MUFA (78.58–84.16%) > SFA (13.60–18.13%) > PUFA (1.01–4.13%). This profile is nutritionally advantageous, as high dietary SFA intake is positively correlated with elevated plasma cholesterol concentrations and increased cardiovascular disease (CVD) risk [28]; while replacing SFA with UFA can counteract its effects on CVD [29]. Among these nine OB, UFA content accounted for 81%, and over 95% of UFA was MUFA. Evidence from clinical trials shows MUFA can benefit cardiovascular patients without side effects [30], as well as diabetes by reducing insulin resistance in participants and improving β-cell function and insulin sensitivity [31]. Moreover, using the (MUFA + PUFA)/SFA ratio is a recommended indicator for dairy fat quality, with high ratio values implying good quality. The (MUFA + PUFA)/SFA ratio for tested OB ranged from 4.51 (H985-O) to 6.32% (G37-O), much higher than that of regular OB [32]. G37-O has prominent advantages over other OB due to its low SFA and high UFA content, as well as the highest (MUFA + PUFA)/SFA ratio.

Oleic acid (C18:1; 77.14–82.03%) and palmitic acid (C16:0; 6.15–7.54%) were the main components of fatty acids in tested OB, followed by stearic acid (C18:0; 1.43–4.37%), linoleic acid (C18:2; 1.04–4.13%), and behenic acid (C22:0; 2.30–3.19%), which is consistent with results observed in the HOP fatty acid profile [33]. High-oleic acid content was a marked characteristic in fatty acid composition for these OB, which is related to peanut cultivars and their inherited traits. J16-O, G37-O, Z215-O, and J572-O showed similar high-oleic acid content, with J6-O, H985-O, and K1715-O showing low oleic acid content (below 80%). High-oleic acid content was associated with postprandial oleoylethanolamide levels and can reduce appetite and suppress energy intake by influencing metabolic and reward systems [34]. Therefore, OB rich in oleic acid represents promising natural ingredients for developing functional foods aimed at weight management and metabolic health.

### 3.4. Tocopherols Composition

Tocopherols are important endogenous antioxidants that can inhibit the internal and external oxidation of vegetable oil and improve the antioxidant capacity of vegetable oil by inhibiting primary oxidation in free radical chain reactions [1]. Previous studies show tocopherol is an intrinsic component of OB, which forms hydrogen bonds with proteins through its own phenolic head group [35]. Therefore, clarifying tocopherol profile and content helps contribute to studies on OB composition, properties, and nutritional value.

Significant quantities of tocopherols (14.83–27.74 mg/100 g) were found in these HOP OB (Table 4), especially in G37-O, H985-O, and Y65-O, whose total tocopherol concentration reached 25 mg/100 g or more. Concentrations of tocopherol isomers analyzed (α-, β-, γ-, and δ-) differed statistically (*p* < 0.05) for both isomers and cultivars. α- and γ-tocopherol were the major tocopherols in the tested OB, which were between 5.07 and 12.59 mg/100 g and 6.36 to 14.81 mg/100 g, respectively. This was consistent with results from previous studies on the composition of tocopherols in peanut oil, where α- and γ-tocopherols were the predominant homologues [36,37]. These differed from most OB (Y37-O, Y65-O, J16-O, J572-O, J6-O, G37-O, and K1715-O), in which γ-tocopherol was the most abundant tocopherol, and Z215-O and H985-O showed the highest α-tocopherol content among the four isomers. This feature can be used as an indicator to identify and characterize different OB cultivars. H985-O, Y65-O, and G37-O contain rich α-tocopherol and γ-tocopherol, meaning a high nutritional and clinical value for these three OB [38]. The content of β-tocopherol and δ-tocopherol in all samples was relatively low, ranging from 0.31 to 0.81 mg/100 g and 1.23 to 1.76 mg/100 g, respectively. Similar results of lower content of β-tocopherol and δ-tocopherol can be seen in reports on OB for other plant seeds, such as Echium plantagineum seed [39], oat [40], and rice bran [41]. Generally, tocopherols in these OB are comprehensive, high in content, and have strong application potential in antioxidation and nutritional fortification.

### 3.5. Amino Acid Composition

As shown in Table 5, the eight essential amino acids (EAA) and eight non-essential amino acids (NEAA) of OB extracted from these nine cultivar peanuts were comprehensively analyzed. Significant inter-cultivar variations were observed in the content of individual amino acids. The amino acid profile of OB covered the full range of EAA and HAA, wherein leucine (0.47–1.41 mg/100 g), phenylalanine (0.43–1.22 mg/100 g), and valine (0.42–1.06 mg/100 g) content were relatively high. Leucine was the most abundant amino acid in both EAA and HAA (0.47–1.41 mg/100 g), whereas histidine (0.10–0.51 mg/100 g) and methionine (0.10–0.16 mg/100 g) were relatively lacking for EAA and HAA, respectively. Lysine, the first limiting amino acid of peanuts, was widely enriched in all OB tested, ranging from 0.25 mg/100 g (J16-O) to 1.10 mg/100 g (Y37-O). In most cases, Y37-O and J16-O showed the highest and lowest amino acid content, resulting in the highest and lowest values for TEAA, THAA, and TAA of 7.11 and 2.40 mg/100 g, 6.23 and 2.23 mg/100 g, 16.78 and 4.82 mg/100 g, respectively. All other OB, including Y65-O, J572-O, J6-O, Z215-O, G37-O, H985-O, and K1715-O, showed a similar amino acid profile with their TEAA, THAA, and TAA ranging from 3.21 to 4.08 mg/100 g, 2.95 to 3.83 mg/100 g, and 6.56 to 8.69 mg/100 g. TEAA/TAA and TEAA/TNEAA were typically used to evaluate protein composition quality. The two quality indicators of OB tested ranged from 42.30% to 49.70% and 73.50% to 99.10%, with both satisfying the ratio of the ideal protein model recommended by the FAO/WHO, with TEAA/TAA ≈ 40% and TEAA/TNEAA ≥ 60% [42].

## 4. Conclusions

The composition of OB from the nine HOPs varied among cultivars. G37-O exhibited a distinct RGB value order, the highest crude protein content, and notable advantages in reducing CVD risk due to its high UFA content and favorable (MUFA + PUFA)/SFA ratio. J572-O, J6-O, Z215-O, and H985-O contained significantly higher fat content than other OB, making them suitable as carriers for oil-soluble active substances. The fatty acid profiles of J16-O, G37-O, Z215-O, and J572-O were characterized by high-oleic acid content, whereas J6-O, H985-O, and K1715-O had relatively lower levels. These HOP OB also contained significant amounts of tocopherols, particularly in G37-O, H985-O, and Y65-O, suggesting high nutritional and clinical value. Y37-O and J16-O showed the highest and lowest amino acid content, respectively, leading to corresponding differences in TEAA, THAA, and TAA. These variations underscore their divergent emulsification performance and nutritional value.

## Figures and Tables

**Table 1:** Quantitative analysis of RGB Color values in OB from nine high-oleic peanut cultivars.

Cultivar	R	G	B	r	g	b
Y37	166.00 ± 1.83 ^b^	169.50 ± 1.29 ^ab^	161.00 ± 1.41 ^bc^	0.3343 ± 0.0020 ^bcde^	0.3413 ± 0.0000 ^a^	0.3242 ± 0.0020 ^abcd^
Y65	175.00 ± 4.08 ^ab^	177.75 ± 4.35 ^ab^	167.75 ± 4.11 ^abc^	0.3362 ± 0.0012 ^bc^	0.3414 ± 0.0005 ^a^	0.3222 ± 0.0016 ^cd^
J16	175.75 ± 9.95 ^a^	179.75 ± 10.37 ^a^	173.25 ± 10.63 ^a^	0.3324 ± 0.0018 ^e^	0.3399 ± 0.0004 ^a^	0.3276 ± 0.0015 ^a^
J572	174.50 ± 4.51 ^ab^	178.00 ± 4.55 ^a^	170.50 ± 5.07 ^ab^	0.3336 ± 0.0016 ^cde^	0.3403 ± 0.0008 ^a^	0.3259 ± 0.0017 ^ab^
J6	173.75 ± 8.22 ^ab^	178.25 ± 10.01 ^a^	170.25 ± 10.87 ^ab^	0.3328 ± 0.0030 ^de^	0.3413 ± 0.0002 ^a^	0.3258 ± 0.0029 ^ab^
Z215	168.25 ± 2.06 ^ab^	172.50 ± 3.51 ^ab^	163.75 ± 3.86 ^abc^	0.3335 ± 0.0021 ^cde^	0.3419 ± 0.0012 ^a^	0.3245 ± 0.0020 ^abc^
G37	168.25 ± 1.71 ^ab^	167.25 ± 6.18 ^b^	158.50 ± 1.29 ^c^	0.3406 ± 0.0033 ^a^	0.3384 ± 0.0066 ^a^	0.3208 ± 0.0038 ^d^
H985	168.25 ± 8.1 ^ab^	170.75 ± 8.02 ^ab^	162.00 ± 8.04 ^bc^	0.3358 ± 0.0009 ^bcd^	0.3408 ± 0.0010 ^a^	0.3233 ± 0.0004 ^bcd^
K1715	168.50 ± 2.38 ^ab^	170.50 ± 3.11 ^ab^	160.75 ± 3.59 ^bc^	0.3371 ± 0.0013 ^b^	0.3411 ± 0.0012 ^a^	0.3216 ± 0.0020 ^cd^

R: red value; G: green value; B: blue value; r: normalized R; g: normalized G; b: normalized B. Different letters in the same column mean a significant difference (*p* < 0.05).

**Table 2:** Basic composition of OB extracted from different high-oleic peanuts.

Cultivar	Moisture (%, Wet Base)	Crude Fat (%, Dry Basis)	Crude Protein (%, Dry Basis)
Y37	20.83 ± 0.50 ^abc^	76.98 ± 0.74 ^f^	1.44 ± 0.08 ^ab^
Y65	18.00 ± 0.85 ^bc^	88.84 ± 0.67 ^c^	1.49 ± 0.09 ^ab^
J16	18.31 ± 1.34 ^c^	82.36 ± 1.86 ^de^	1.44 ± 0.45 ^ab^
J572	22.00 ± 0.17 ^a^	92.73 ± 0.31 ^a^	1.43 ± 0.16 ^ab^
J6	22.05 ± 0.21 ^a^	91.06 ± 1.13 ^b^	1.12 ± 0.13 ^cd^
Z215	20.50 ± 1.41 ^abc^	91.36 ± 0.43 ^b^	1.24 ± 0.03 ^bc^
G37	24.25 ± 2.03 ^ab^	82.70 ± 0.27 ^d^	1.59 ± 0.08 ^a^
H985	19.40 ± 1.41 ^abc^	93.22 ± 1.28 ^a^	0.89 ± 0.01 ^d^
K1715	21.70 ± 2.12 ^ab^	81.59 ± 1.41 ^e^	0.98 ± 0.01 ^cd^

Different letters in the same column mean a significant difference (*p* < 0.05).

**Table 3:** Fatty acid compositions (%) of OB extracted from different high-oleic peanuts.

Fatty Acid	Y37	Y65	J16	J572	J6	Z215	G37	H985	K1715
C16:0 (Palmitic acid)	6.91 ± 0.02 ^c^	6.21 ± 0.02 ^ef^	6.39 ± 0.02 ^d^	6.26 ± 0.08 ^ef^	6.29 ± 0.08 ^de^	6.15 ± 0.05 ^f^	7.35 ± 0.14 ^b^	7.48 ± 0.03 ^a^	7.54 ± 0.05 ^a^
C18:0 (Stearic acid)	2.95 ± 0.01 ^e^	3.02 ± 0.07 ^e^	3.55 ± 0.02 ^d^	4.37 ± 0.05 ^a^	3.67 ± 0.03 ^cd^	4.23 ± 0.10 ^a^	1.43 ± 0.17 ^f^	3.81 ± 0.06 ^bc^	3.88 ± 0.11 ^b^
C18:1 (Oleic acid)	80.5 ± 0.02 ^e^	80.8 ± 0.05 ^d^	82.03 ± 0.02 ^a^	81.01 ± 0.09 ^d^	79.70 ± 0.08 ^f^	81.25 ± 0.17 ^c^	81.70 ± 0.16 ^b^	78.45 ± 0.04 ^g^	77.14 ± 0.17 ^h^
C18:2 (linoleic acid)	2.33 ± 0.02 ^b^	1.51 ± 0.04 ^c^	1.47 ± 0.06 ^c^	1.55 ± 0.06 ^c^	2.42 ± 0.06 ^b^	1.04 ± 0.10 ^e^	2.33 ± 0.1 ^b^	2.01 ± 0.02 ^c^	4.13 ± 0.04 ^a^
C20:0 (Arachidic acid)	1.48 ± 0.07 ^d^	1.55 ± 0.03 ^cd^	1.63 ± 0.02 ^c^	1.82 ± 0.06 ^b^	1.73 ± 0.05 ^b^	1.79 ± 0.07 ^b^	0.94 ± 0.05 ^e^	1.95 ± 0.03 ^a^	1.82 ± 0.04 ^b^
C20:1 (Eicosenoic acid)	1.64 ± 0.03 ^c^	2.07 ± 0.02 ^b^	1.35 ± 0.02 ^ef^	1.30 ± 0.11 ^f^	1.51 ± 0.04 ^d^	1.41 ± 0.03 ^de^	2.41 ± 0.04 ^a^	1.39 ± 0.04 ^ef^	1.43 ± 0.09 ^de^
C22:0 (Behenic acid)	2.74 ± 0.04 ^d^	3.09 ± 0.03 ^b^	2.42 ± 0.03 ^f^	2.56 ± 0.04 ^e^	2.90 ± 0.03 ^c^	2.65 ± 0.05 ^e^	2.30 ± 0.07 ^h^	3.19 ± 0.03 ^a^	2.82 ± 0.08 ^cd^
C24:0 (Lignoceric acid)	1.33 ± 0.01 ^c^	1.73 ± 0.02 ^a^	1.14 ± 0.03 ^d^	1.11 ± 0.05 ^d^	1.77 ± 0.04 ^a^	1.46 ± 0.10 ^b^	1.46 ± 0.09 ^b^	1.69 ± 0.06 ^a^	1.21 ± 0.05 ^d^
∑SFA	15.40 ± 0.16 ^e^	15.51 ± 0.11 ^e^	15.11 ± 0.02 ^f^	16.11 ± 0.14 ^d^	16.30 ± 0.07 ^c^	16.29 ± 0.06 ^cd^	13.60 ± 0.31 ^h^	18.13 ± 0.07 ^a^	17.29 ± 0.05 ^b^
∑MUFA	82.20 ± 0.05 ^e^	82.80 ± 0.07 ^c^	83.32 ± 0.04 ^b^	82.30 ± 0.19 ^e^	81.20 ± 0.04 ^f^	82.67 ± 0.15 ^d^	84.16 ± 0.2 ^a^	79.85 ± 0.08 ^g^	78.58 ± 0.08 ^h^
∑UFA	84.50 ± 0.08 ^c^	84.40 ± 0.11 ^c^	84.80 ± 0.02 ^b^	83.81 ± 0.13 ^d^	83.61 ± 0.07 ^e^	83.71 ± 0.06 ^de^	86.40 ± 0.18 ^a^	81.86 ± 0.07 ^h^	82.71 ± 0.06 ^f^
(MUFA + PUFA)/SFA	5.48 ± 0.49 ^c^	5.41 ± 0.04 ^c^	5.59 ± 0.01 ^b^	5.19 ± 0.05 ^d^	5.11 ± 0.02 ^d^	5.13 ± 0.02 ^d^	6.32 ± 0.14 ^a^	4.51 ± 0.02 ^f^	4.78 ± 0.02 ^e^

SFA: total saturated fatty acid; MUFA: total monounsaturated fatty acid; PUFA: total polyunsaturated fatty acid; UFA: total unsaturated fatty acid. Different letters in the same row mean a significant difference (*p* < 0.05).

**Table 4:** Tocopherols of OB extracted from different high-oleic peanuts (mg/100 g).

Cultivar	α-Tocopherol	β-Tocopherol	γ-Tocopherol	δ-Tocopherol	Total Tocopherols
Y37	6.84 ± 0.27 ^e^	0.59 ± 0.03 ^cd^	8.89 ± 0.08 ^d^	1.69 ± 0.05 ^b^	18.02 ± 0.27 ^e^
Y65	10.59 ± 0.24 ^b^	0.71 ± 0.16 ^ab^	13.21 ± 0.23 ^b^	1.43 ± 0.06 ^c^	25.95 ± 0.67 ^c^
J16	5.07 ± 0.10 ^h^	0.31 ± 0.02 ^e^	8.02 ± 0.17 ^e^	1.42 ± 0.04 ^c^	14.83 ± 0.23 ^h^
J572	6.29 ± 0.13 ^g^	0.49 ± 0.01 ^d^	7.22 ± 0.19 ^f^	1.23 ± 0.09 ^d^	15.24 ± 0.34 ^g^
J6	6.77 ± 0.24 ^ef^	0.63 ± 0.05 ^bc^	7.33 ± 0.29 ^f^	1.59 ± 0.02 ^bc^	16.33 ± 0.21 ^f^
Z215	6.63 ± 0.19 ^f^	0.52 ± 0.08 ^cd^	6.36 ± 0.12 ^g^	1.42 ± 0.07 ^c^	14.94 ± 0.17 ^h^
G37	10.19 ± 0.13 ^c^	0.81 ± 0.11 ^a^	14.81 ± 0.30 ^a^	1.92 ± 0.05 ^a^	27.74 ± 0.85 ^a^
H985	12.59 ± 0.07 ^a^	0.72 ± 0.16 ^ab^	11.41 ± 0.26 ^c^	1.58 ± 0.02 ^bc^	26.31 ± 0.51 ^b^
K1715	7.89 ± 0.21 ^d^	0.55 ± 0.02 ^cd^	8.09 ± 0.11 ^e^	1.76 ± 0.01 ^ab^	18.30 ± 0.33 ^d^

Different letters in the same column mean a significant difference (*p* < 0.05).

**Table 5:** Amino acid composition (mg/100 g) of OB extracted from different high-oleic peanuts.

Amino Acids	Y37	Y65	J16	J572	J6	Z215	G37	H985	K1715
Essential amino acids									
Threonine	0.86 ± 0.01 ^a^	0.54 ± 0.02 ^d^	0.35 ± 0.02 ^g^	0.62 ± 0.01 ^c^	0.52 ± 0.03 ^e^	0.54 ± 0.10 ^d^	0.64 ± 0.03 ^b^	0.48 ± 0.00 ^f^	0.55 ± 0.02 ^d^
Valine *	1.06 ± 0.01 ^a^	0.58 ± 0.01 ^c^	0.42 ± 0.02 ^f^	0.64 ± 0.00 ^b^	0.56 ± 0.01 ^d^	0.58 ± 0.01 ^c^	0.64 ± 0.01 ^b^	0.52 ± 0.02 ^e^	0.58 ± 0.02 ^c^
Methionine *	0.13 ± 0.03 ^b^	0.12 ± 0.03 ^b^	0.10 ± 0.01 ^b^	0.13 ± 0.01 ^b^	0.16 ± 0.01 ^a^	0.15 ± 0.02 ^a^	0.14 ± 0.06 ^a^	0.12 ± 0.03 ^b^	0.13 ± 0.01 ^b^
Isoleucine *	0.82 ± 0.02 ^a^	0.44 ± 0.00 ^e^	0.28 ± 0.02 ^g^	0.49 ± 0.02 ^c^	0.42 ± 0.01 ^e^	0.44 ± 0.00 ^d^	0.52 ± 0.02 ^b^	0.38 ± 0.02 ^f^	0.45 ± 0.00 ^d^
Leucine *	1.41 ± 0.01 ^a^	0.76 ± 0.00 ^f^	0.47 ± 0.00 ^h^	0.84 ± 0.02 ^c^	0.74 ± 0.02 ^f^	0.78 ± 0.06 ^d^	0.87 ± 0.02 ^b^	0.67 ± 0.01 ^g^	0.78 ± 0.01 ^e^
Phenylalanine *	1.22 ± 0.02 ^a^	0.56 ± 0.01 ^cd^	0.43 ± 0.01 ^f^	0.6 ± 0.01 ^b^	0.55 ± 0.00 ^d^	0.56 ± 0.01 ^cd^	0.62 ± 0.00 ^b^	0.52 ± 0.03 ^e^	0.56 ± 0.03 ^c^
Lysine	1.10 ± 0.01 ^a^	0.40 ± 0.01 ^d^	0.25 ± 0.01 ^h^	0.47 ± 0.03 ^b^	0.38 ± 0.00 ^f^	0.40 ± 0.03 ^e^	0.46 ± 0.01 ^b^	0.36 ± 0.04 ^g^	0.43 ± 0.03 ^c^
Histidine	0.51 ± 0.00 ^a^	0.17 ± 0.02 ^c^	0.10 ± 0.03 ^d^	0.19 ± 0.00 ^c^	0.17 ± 0.03 ^c^	0.17 ± 0.03 ^c^	0.19 ± 0.03 ^b^	0.16 ± 0.04 ^c^	0.18 ± 0.05 ^c^
TEAA	7.11 ± 0.04 ^a^	3.57 ± 0.01 ^e^	2.40 ± 0.05 ^h^	3.98 ± 0.04 ^c^	3.50 ± 0.06 ^f^	3.62 ± 0.17 ^d^	4.08 ± 0.07 ^b^	3.21 ± 0.11 ^g^	3.66 ± 0.09 ^d^
Non-essential amino acids									
Aspartate	1.76 ± 0.02 ^a^	0.48 ± 0.03 ^f^	0.31 ± 0.00 ^h^	0.56 ± 0.04 ^c^	0.50 ± 0.00 ^e^	0.52 ± 0.01 ^d^	0.59 ± 0.03 ^b^	0.44 ± 0.03 ^g^	0.56 ± 0.01 ^c^
Serine	0.90 ± 0.01 ^a^	0.42 ± 0.03 ^e^	0.26 ± 0.01 ^h^	0.47 ± 0.01 ^c^	0.40 ± 0.02 ^f^	0.43 ± 0.03 ^d^	0.50 ± 0.01 ^b^	0.37 ± 0.03 ^g^	0.44 ± 0.01 ^c^
Glutamate	2.40 ± 0.01 ^a^	0.65 ± 0.01 ^e^	0.42 ± 0.01 ^g^	0.74 ± 0.01 ^c^	0.72 ± 0.02 ^d^	0.71 ± 0.03 ^d^	0.78 ± 0.00 ^b^	0.62 ± 0.00 ^f^	0.76 ± 0.04 ^c^
Glycine	0.88 ± 0.01 ^a^	0.56 ± 0.01 ^e^	0.37 ± 0.03 ^g^	0.65 ± 0.03 ^c^	0.55 ± 0.03 ^e^	0.60 ± 0.02 ^d^	0.68 ± 0.01 ^b^	0.50 ± 0.02 ^f^	0.58 ± 0.02 ^d^
Alanine *	0.93 ± 0.03 ^a^	0.58 ± 0.04 ^e^	0.37 ± 0.00 ^h^	0.65 ± 0.02 ^c^	0.56 ± 0.01 ^f^	0.60 ± 0.00 ^de^	0.67 ± 0.01 ^b^	0.50 ± 0.01 ^g^	0.60 ± 0.00 ^d^
Tyrosine *	0.66 ± 0.05 ^a^	0.31 ± 0.00 ^c^	0.16 ± 0.05 ^f^	0.36 ± 0.00 ^b^	0.26 ± 0.00 ^d^	0.30 ± 0.00 ^c^	0.36 ± 0.01 ^b^	0.24 ± 0.03 ^e^	0.31 ± 0.01 ^c^
Arginine	1.40 ± 0.01 ^a^	0.47 ± 0.02 ^e^	0.28 ± 0.01 ^g^	0.54 ± 0.00 ^c^	0.48 ± 0.02 ^d^	0.50 ± 0.01 ^d^	0.57 ± 0.04 ^b^	0.42 ± 0.02 ^f^	0.52 ± 0.03 ^c^
Proline	0.74 ± 0.02 ^a^	0.42 ± 0.01 ^c^	0.25 ± 0.02 ^h^	0.41 ± 0.01 ^c^	0.33 ± 0.01 ^f^	0.40 ± 0.01 ^d^	0.46 ± 0.02 ^b^	0.26 ± 0.02 ^g^	0.36 ± 0.02 ^e^
Tryptophan *	n.d.								
TNEAA	9.67 ± 0.09 ^a^	3.89 ± 0.05 ^f^	2.42 ± 0.10 ^i^	4.38 ± 0.13 ^c^	3.80 ± 0.08 ^g^	4.06 ± 0.05 ^e^	4.61 ± 0.05 ^b^	3.35 ± 0.08 ^h^	4.13 ± 0.07 ^d^
TAA	16.78 ± 0.13 ^a^	7.46 ± 0.10 ^f^	4.82 ± 0.26 ^i^	8.36 ± 0.19 ^c^	7.30 ± 0.13 ^g^	7.68 ± 0.05 ^e^	8.69 ± 0.17 ^b^	6.56 ± 0.10 ^h^	7.79 ± 0.15 ^d^
TEAA/TAA (%)	42.30 ± 0.14 ^f^	47.80 ± 0.12 ^c^	49.70 ± 0.31 ^a^	47.60± 0.30 ^d^	47.94± 0.20 ^c^	47.13± 0.07 ^d^	46.90 ± 0.21 ^e^	48.93± 0.11 ^b^	46.98± 0.16 ^e^
TEAA/TNEAA (%)	73.50 ± 0.45 ^f^	91.70 ± 0.47 ^c^	99.10 ± 1.23 ^a^	90.80± 1.08 ^d^	92.10± 0.74 ^c^	89.16± 0.25 ^d^	88.50± 0.40 ^e^	95.82± 0.43 ^b^	88.61± 0.57 ^e^

*: hydrophobic amino acids; n.d., not detected; TEAA: total essential amino acids; TNEAA: total non-essential amino acids; TAA: total amino acids. Different letters in the same row mean a significant difference (*p* < 0.05).

## Data Availability

The original contributions presented in this study are included in the article. Further inquiries can be directed to the corresponding author.

## Abbreviations

The following abbreviations are used in this manuscript:

HOP	High-oleic peanut
OB	Oil body
Y37	Yuhua 37
Y65	Yuhua 65
J16	Jihua 16
J572	Jihua 572
J6	Jiyou 6 hao
Z215	Zhonghua 215
G37	Guihua 37
H985	Huayu 985
K1715	Kainong 1715
Y37-O	OB extracted from Y37 and other samples were named in the same way
CAD	Cardiovascular
SFA	saturated fatty acid
MUFA	monounsaturated fatty acid
PUFA	polyunsaturated fatty acid
UFA	unsaturated fatty acid
HAA	hydrophobic amino acids
TEAA	total essential amino acids
TNEAA	total nonessential amino acids
TAA	total amino acids
O/L	oleic to linoleic acid ratio
